# Theoretical Study of the Transpore Velocity Control of Single-Stranded DNA

**DOI:** 10.3390/ijms150813817

**Published:** 2014-08-11

**Authors:** Weixin Qian, Kentaro Doi, Satoshi Uehara, Kaito Morita, Satoyuki Kawano

**Affiliations:** Department of Mechanical Science and Bioengineering, Graduate School of Engineering Science, Osaka University, 1-3 Machikaneyama, Toyonaka, Osaka 560-8531, Japan; E-Mails: qian@bnf.me.es.osaka-u.ac.jp (W.Q.); doi@me.es.osaka-u.ac.jp (K.D.); uehara@paris.ifs.tohoku.ac.jp (S.U.); morita@bnf.me.es.osaka-u.ac.jp (K.M.)

**Keywords:** ssDNA, micro/nanofluidics, langevin dynamics simulation, transpore dynamics, coarse-graining

## Abstract

The electrokinetic transport dynamics of deoxyribonucleic acid (DNA) molecules have recently attracted significant attention in various fields of research. Our group is interested in the detailed examination of the behavior of DNA when confined in micro/nanofluidic channels. In the present study, the translocation mechanism of a DNA-like polymer chain in a nanofluidic channel was investigated using Langevin dynamics simulations. A coarse-grained bead-spring model was developed to simulate the dynamics of a long polymer chain passing through a rectangular cross-section nanopore embedded in a nanochannel, under the influence of a nonuniform electric field. Varying the cross-sectional area of the nanopore was found to allow optimization of the translocation process through modification of the electric field in the flow channel, since a drastic drop in the electric potential at the nanopore was induced by changing the cross-section. Furthermore, the configuration of the polymer chain in the nanopore was observed to determine its translocation velocity. The competition between the strength of the electric field and confinement in the small pore produces various transport mechanisms and the results of this study thus represent a means of optimizing the design of nanofluidic devices for single molecule detection.

## 1. Introduction

The high-speed reading of deoxyribonucleic acid (DNA) sequences is an important means of elucidating complete genetic sequences, and may enable the development of new medical treatments [1,2]. Recently, novel DNA and ribonucleic acid (RNA) sequencing technologies have been developed. Among these, nanopore sequencing devices are one of the most significant issues and represent an emerging non-optical process for high-throughput single-molecule detection [1,2,3,4], in which individual nucleobases are identified by measuring transpore ionic current blockade [5,6,7] or transverse tunneling current [8,9,10,11] during the transport of single-stranded DNA (ssDNA) through a nanometer-sized gap. Understanding biological polymer transport phenomena is a crucial issue in the development of DNA sequencing techniques, as well as in the study of many of the physical properties of polymers [12], and both the theoretical [13,14,15,16,17] and experimental [18,19,20,21,22,23,24,25,26,27,28] aspects of polymer translocation through nanopores have been widely studied. Computational studies have provided particularly valuable insights into the physics of transport within confined micro/nanochannels and previous works have examined the variation of translocation time with polymer chain length [13,14,15,19,21,29,30,31,15,19,21,29], pore dimensions [31], driving force [15,19,21,31], sequences and secondary structures [21,22,32], polymer–pore interactions [21,22,23,33], and polymer configurations [15,34]. Table 1 lists the various nanopore devices and polymers used in the pioneering research studies investigating these subjects with the aim of achieving an advanced DNA sequencer.

**Table 1:** Nanopore devices and deoxyribonucleic acid (DNA)/ribonucleic acid (RNA) samples used in published experimental studies.

Group	Pore Type	Diameter (nm)	Length (nm)	Voltage (mV)	Voltage/Length (×10^6^ V/m)	Polymer Length (bp or nt)	Polymer Type
Kasianowicz *et al.* [5]	α-HL	1.3	5.2	120	23	150	ssDNA, ssRNA *
Meller *et al.* [18,19,20]	α-HL	1.3	5.2	50–300	9.6–58	5–100	ssDNA
Butler *et al.* [35]	MspA	1	10	140, 180	14, 18	50	ssDNA
Wendell *et al.* [36]	Phi29	3.3	7.5	40, 75	5.3, 10	5.5 k	dsDNA
Franceschini *et al.* [37]	ClyA	7.8	13	100	7.7	290 bp, 51 nt	dsDNA, ssDNA
Li *et al.* [21]	SiN	3, 10	5–10	60, 120	6–24	3–10 k	dsDNA
Storm *et al.* [22]	SiN	10	20	100–600	5–30	10–97 k	dsDNA
Skinner *et al.* [24]	SiN	10	20	100–600	5–30	10–30 k	dsDNA, dsRNA, ssRNA *
Tsutsui *et al.* [26]	SiN	50	200	1000	5	48.5 k	dsDNA
Fologea *et al.* [27,28]	SiN	10	10, 280	120	0.43, 12	3 k	dsDNA, ssDNA
Schneider *et al.* [25]	Graphene	22	0.3	200	670	48.5 k	dsDNA

* ssRNA denotes poly(A), poly(C), and poly(U).

Sung and Park [13] and Muthukumar [14] studied the passage of single polymer molecules through the pores of a membrane during diffusion across a free energy barrier due to chemical potential differences. Both groups modeled the stochastic processes associated with the transport of long polymers based on the Fokker–Planck equation and were able to predict a scaling law describing translocation time, τ, as a function of polymer length, *N*. Storm *et al.* [22] and Skinner *et al.* [24] investigated the translocation of double-stranded DNA (dsDNA) through silicon nitride (SiN) nanopores that were 10 nm in diameter and 30 nm thick. They also identified that a power law best described the relationship between τ and the polymer length, such that τ~*N*^1.27^. The use of ultrathin nanopores (0.3 nm thick) fabricated within a graphene monolayer [25] is known to result in a slightly larger τ value than that obtained using SiN nanopores [21,22] and it has been suggested that these small pores as well as interactions with the graphene result in the slower translocation. This phenomenon has also been investigated on the basis of Langevin dynamics simulations [31,34]. In other works, Meller *et al.* [19] studied the translocation of ssDNA through a biological α-hemolysin (α-HL) nanopore and determined that the translocation velocity of short polymers exhibited a significant dependence on the length of the polymer, whereas there was no dependence in the case of long polymers. The engineered Mycobacterium smegmatis porin A (MspA) [35] and phi29 [36] protein nanopores were found to allow the translocation of ssDNA and dsDNA with remarkable stability against environmental stresses. It is indicated that an engineered DNA transporter is able to recognize and chaperone the specific DNA molecule across a biological membrane, making a further step for the application of a nanofluidic platform [37]. It was also found that, during forced translocation in narrow pores, the scaling exponents depended on the driving force, *F*, based on the relationship τ~*F*^−1^ [15,30,31]. Although the hydrodynamic effects on polymer chains appear to account for part of the force counteracting external forces [13,29], these effects seem to make only a minor contribution to the transport of DNA, since it has a large number of charges and small surface areas in comparison to other polymer particles [38,39,40]. In particular, the electrokinetic transport of DNA passing through very narrow spaces is predominantly affected by collisions with channel walls [41].

**Figure 1:**
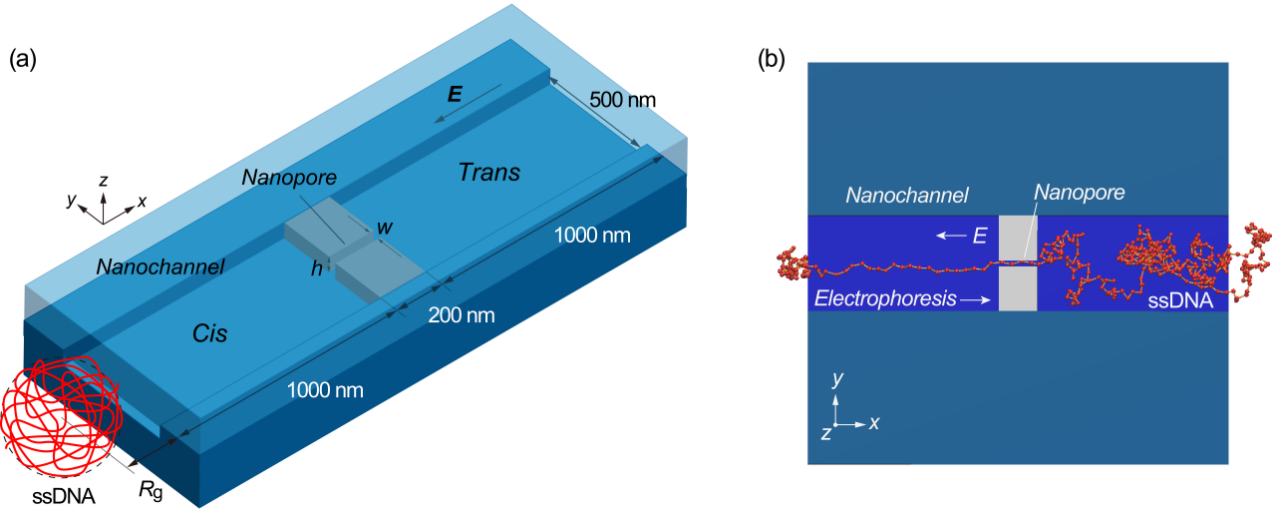
(**a**) Schematic illustration of a rectangular nanochannel used in Langevin dynamics simulations, in which a nanopore is embedded in the nanochannel. In the three-dimensional model, the width (*w*) and height (*h*) of a 200 nm long nanopore are varied as simulation parameters in a nanochannel of 2200 nm in length, 500 nm in width, and *h* in height. The center of mass of single-stranded DNA (ssDNA) is initially located at a distance of the radius of gyration (*R*_g_ = 300 nm) away from the nanochannel entrance where the *x-* and *y-*coordinate of the mass center are in coincidence with the center of nanochannel; and (**b**) An illustration showing the coarse-grained bead-spring model of ssDNA in the simulations to assess the electrokinetic transport dynamics and to optimize the structure of the nanofluidic channel for single molecule sensing.

We are interested in a long polymer translocation mechanism in micro/nanochannels and nanopores [42,43,44] under the effects of nonuniform electric fields, since such mechanisms have not yet been sufficiently elucidated. In the present study, we attempt to gain a better understanding of the translocation mechanism of a DNA-like polymer chain, equivalent to 48 × 10^3^ nucleotides (48 knt), penetrating a solid-sate nanopore in the presence of nonuniform electric fields, as illustrated in Figure 1. The cross-section of the nanopore is expected to play an important role in terms of controlling the translocation process. While nanopores embedded in nanochannels were supposed to be effective to slow down the transpore velocity of ssDNA [42,44], the mechanism has remained to be clarified. Herein, focusing on the multiply-connected nanofluidic channels, the retardation process and its advantage are discussed from a theoretical point of view. As part of this work, we develop a coarse-grained ssDNA model [16,45,46] and perform Langevin dynamics simulations of ssDNA transport under nonuniform electric fields in a rectangular nanochannel containing a nanopore with various cross-sections [47,48,49,50,51], where the electric fields are calculated for the cross-sections, ranging from 20 × 20 to 50 × 50 nm^2^. The results allow a visual analysis of the electrokinetic transport dynamics of ssDNA chains and allow us to determine the most suitable morphology for nanofluidic flow channels for single molecule detection. Furthermore, the simulation results are clearly understood by a theoretical model in the framework of the Langevin equation. Consequently, a relationship among the electrokinetic transport of ssDNA, pore dimensions, and multiply-connected structures of the nanofluidic channel are clarified and a desirable design to control the translocation velocity is concluded.

## 2. Results and Discussion

### 2.1. Validation of the Coarse-Grained Single-Stranded DNA (ssDNA) Model

**Figure 2:**
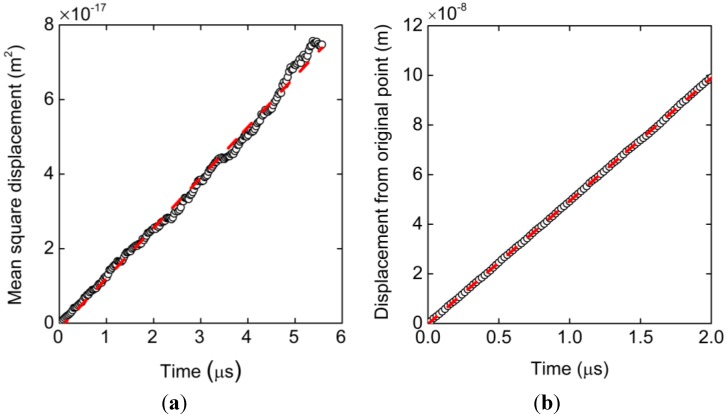
(**a**) Mean square displacement of the bead-spring model resulting from 90 simulation runs and (**b**) displacement of the mass center of the bead-spring chain under an electric field of 1 × 10^6^ V/m obtained from 270 simulation runs. Each result is well fitted with straight lines by the least-squares method. The slope of plot (**a**) corresponds to the diffusion coefficient while that of (**b**) is the velocity that translates to the electrophoretic mobility.

As a result of the Langevin dynamics simulation, Figure 2a shows the mean square displacement of the center of mass of the ssDNA model as a function of time. This plot represents the average of results from 90 simulations at each data point and is clearly linear. The associated diffusion coefficient, *D*, can be obtained according to the Einstein relation and is calculated to be 2.25 × 10^−12^ m^2^/s. Figure 2b presents a plot of the distance from the origin to the center of mass under an external electric field of 1 × 10^6^ V/m, in which each data point is the average of 270 simulations. The electrophoretic mobility calculated from the ratio of the velocity values to the applied electric field strengths is μ = 2.87 × 10^−8^ m^2^/Vs. These two results are in close agreement with the experimental data described in the methodology (Section 3) [52]. From the viewpoint of diffusivity and electrophoretic mobility, the present parameter set is therefore acceptable when assessing the electrokinetic transport of ssDNA.

### 2.2. Langevin Dynamics Simulations

**Figure 3:**
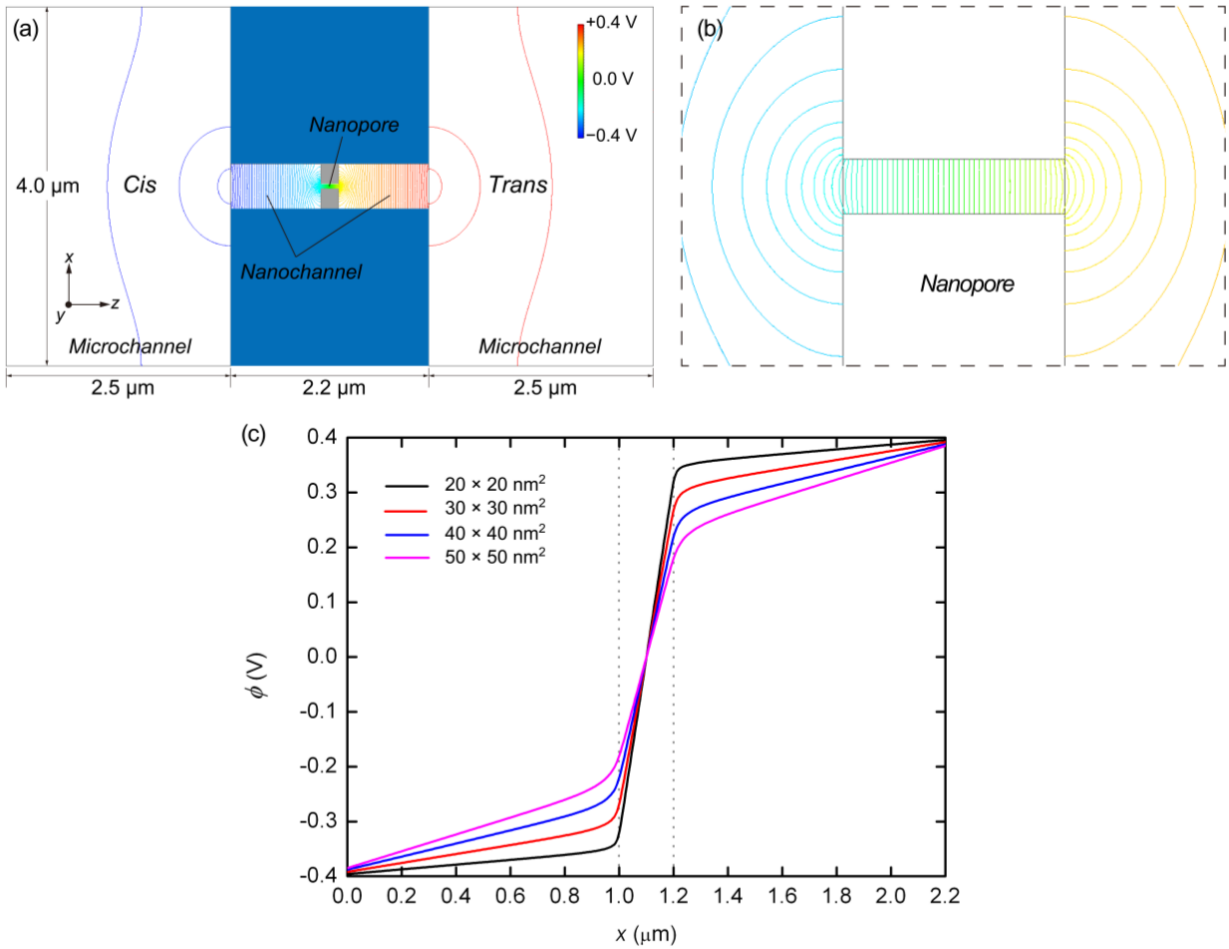
(**a**) Electrostatic potential resulting from FEM analysis for the system including microchannel, nanochannel, and nanopore whose cross-section is 50 × 50 nm^2^; (**b**) Magnified view of (**a**) focusing onnear the nanopore;and (**c**) Electrostatic potentials, ϕ, for nanopores of cross-section 20 × 20, 30 × 30, 40 × 40, and 50 × 50 nm^2^, resulting from three-dimensional FEM analyses. The entrance and exit of the nanopores are indicated by the dotted lines.

As shown in Figure 3, electrostatic potentials across the microchannel, nanochannel, and nanopore are determined from the finite element method (FEM) analysis [53], in which the potential curves extracted along the central axis are presented for several nanopore cross-sections. It is found that the slope of the electrostatic potential becomes steeper in the narrower channels, as shown in Figure 3a,b. A large drop in the potential at the nanopore suppresses the potential difference outside the nanopore. As can be seen from Figure 3c, the electric field strength increases as the cross-sectional area of the nanopore is reduced. The electric field strengths calculated along the central axis of the nanochannel and nanopore with various cross-sections are also summarized in Table 2. In the previous experimental studies as summarized in Table 1, as well as in numerical analyses, other researchers have also found that strong electric fields are associated with nanopores [54]. Some publications note that such a strong electric field tends to be proportional to the value of (*d*_pore_/*d*_eff_)^2^, where *d*_pore_ is the diameter of the pore and *d*_eff_ is the effective diameter outside the pore [54,55]. Our computational results also agree with the potential drop resulting from variations in the nanopore cross-section.

**Table 2:** Electric field and ssDNA transpore properties in the nanochannel and nanopore.

Pore Size (nm^2^)	*E*_channel_ (V/m)	*E*_pore_ (V/m)	*N*_pore_	Δ*N*/Δ*x* (m^−1^)	*v*_channel_ (mm/s)	*v*_pore_ (mm/s)
30 × 30	8.2 × 10^4^	2.7 × 10^6^	6.48	1.30 × 10^9^	2.3	1.3
40 × 40	1.2 × 10^5^	2.2 × 10^6^	6.81	9.64 × 10^8^	3.3	1.1
50 × 50	1.5 × 10^5^	1.9 × 10^6^	9.29	7.45 × 10^8^	4.3	1.2

**Figure 4:**
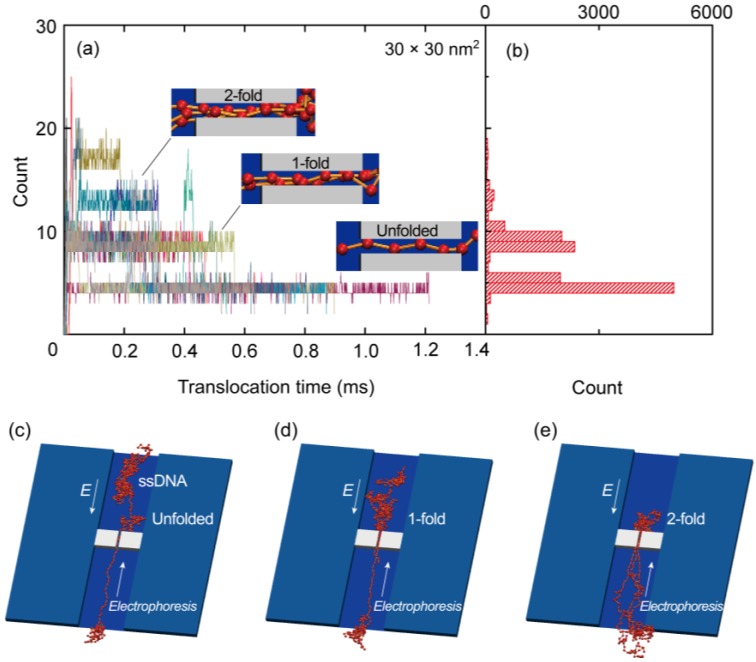
(**a**) Time series data, where each run is presented by color variation; (**b**) histogram of the number of beads in a 30 × 30 nm^2^ cross-section nanopore, obtained from 20 runs of the Langevin dynamics simulation; A leading head enters the pore at *t* = 0 sand the time elapsed is recorded until the end bead leaves the pore. Illustrations of the entire ssDNA chain are also presented, showing (**c**) unfolded; (**d**) 1-fold; and (**e**) 2-fold forms.

**Figure 5:**
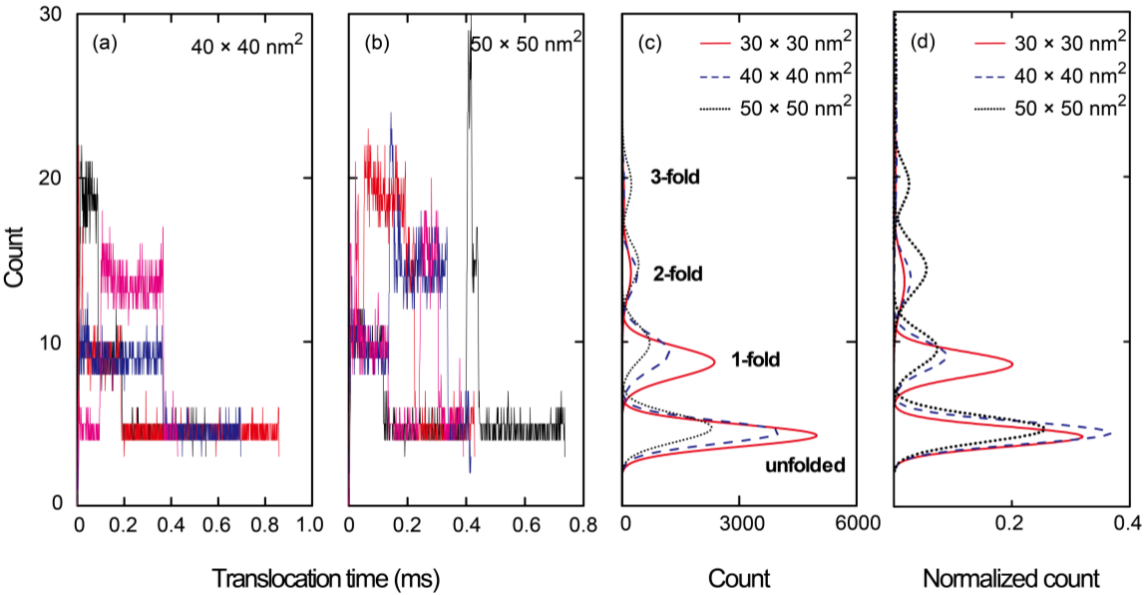
Time series data indicating the number of beads in nanopores of cross-section (**a**) 40 × 40 and (**b**) 50 × 50 nm^2^, in which only four typical data are presented by color variation in each case; (**c**) fitted distribution resulting from the complete data acquired from 20 simulations for each condition; and (**d**) the normalized distribution of (**c**).

As shown in Figure 4a, we also ascertained the number of beads along a 200 nm long nanopore during the simulations. In this figure, the entire data set resulting from 20 simulation runs for a 30 × 30 nm^2^ cross-section nanopore is presented. At *t* = 0 s, a leading bead entered the pore, at which point the elapsed time was tracked until the end of the chain left the pore. The distribution of bead numbers seems to be discretized at several specific numerical values. As can be seen in the insets to this figure, which show illustrations of the nanopore, the discretized numbers correspond to specific folded structures of the polymer chain. Sufficiently uncoiled ssDNA chains tend to pass through the nanopore in an unfolded form and therefore, the translocation time is relatively long. In contrast, coiled chains adopt folded forms in the pore, resulting in shorter translocation periods. Figure 4b presents a summary of the data in Figure 4a in the form of a histogram. The highest peak in this plot corresponds to an unfolded structure, while the second and third highest peaks equate to 1- and 2-fold forms, respectively. More detailed illustrations of the unfolded, 1-fold, and 2-fold forms at the nanopore are also presented in Figure 4c–e, respectively. Figure 5a,b show the results for 40 × 40 and 50 × 50 nm^2^ cross-section nanopores, respectively, where four typical samples are presented by color variations. The time series data in the plots apparently fluctuate with increasing cross-sectional areas. The larger the cross-sections become, the more frequently the ssDNA will change its configuration, thus producing multifold forms in the pore. As a result, the distribution of multifold-structures increases as the cross-sectional area increases. The fitted distributions obtained from 20 simulation runs for each condition are summarized in Figure 5c. The concentrations of electric charges resulting from the folded structures increase the translocation speed due to the associated strong electric force. Although we also performed simulations for a 20 × 20 nm^2^ cross-section nanopore, the electric field outside a nanopore of this size was evidently too weak to introduce the ssDNA into the pore. This result implies that an excessively small pore will require a long period of time to attract charged molecules to it. A weak electric field outside the pore, as is produced in the case of an overly small pore, is therefore disadvantageous for the polymer chain to overcome the entropic barriers at interconnections in the channel, because the large difference of cross-sections requires strong force to uncoil a coiled structure to introduce it into the nanopore [56,57]. Figure 5d presents a normalized version of the distribution data in Figure 5c. Comparing the three cross-sections, it is evident that multifold-structures become prominent as the cross-sectional area increases. With respect to single-molecule detection, it is desirable to maintain unfolded configurations for as long as possible to slow down the translocation speed. Thus, the 30 × 30 nm^2^ cross-section pore is suggested to be the most suited to the sequential transport of ssDNA molecules.

**Figure 6:**
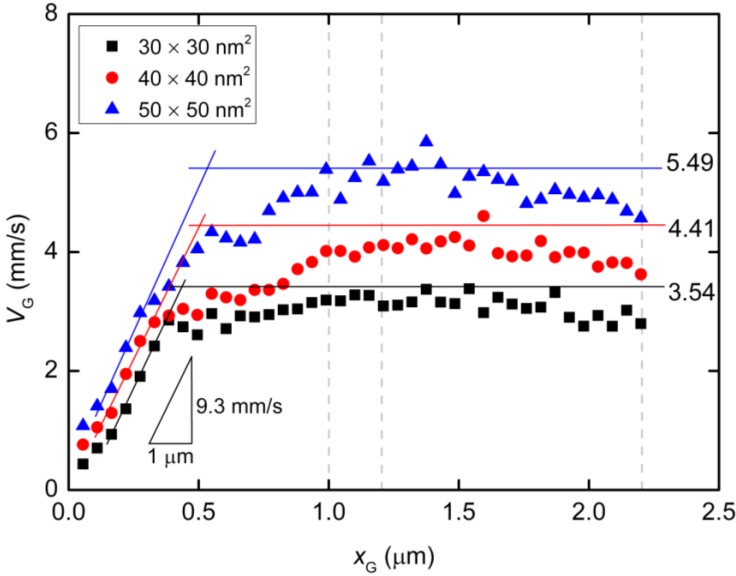
Velocity profile of the centers of mass of ssDNA chains passing through nanopores of cross-section 30 × 30, 40 × 40, and 50 × 50 nm^2^, in which *x*_G_ is the position of mass center measured from the nanochannel entrance along the *x*-axis. The results of theoretical calculations using Equations (4) and (5) are shown as solid lines. The start and end of the nanopore are situated at *x*_G_ = 1.0 and 1.2 μm and the end of the nanochannel is at *x*_G_ = 2.2 μm, all of which are indicated by dashed lines.

For deep understanding of the electrokinetic transport phenomena in the nanofluidic device, the simulation results are analyzed by a theoretical model of the Langevin equation as also described in detail in the methodology section. Particularly, a relationship between the translocation velocity and the pore size attracts most of our interests. Figure 6 shows velocity profiles of the mass centers of the ssDNA for the three cases presented in Figure 5, in which *x*_G_ denotes the position of mass center along the *x*-axis measured from the nanochannel entrance (2200 nm in total), and the nanopore is located from *x*_G_ = 1000 to 1200 nm. In overdamped Langevin dynamics simulations, the velocity of a particle is directly proportional to the force on it, as theoretically described in the next section. For each cross-section, the velocity linearly increased until the mass center reached an *x*_G_ value of approximately 500 nm, at which point the leading bead moved into the stronger field while the remainder of the chain was still in front of the nanochannel entrance. Therefore, the number of beads in the nanochannel increased in a stepwise fashion over time. According to the Langevin equation, the equation of motion of the mass center along the pore axis may be roughly expressed by:

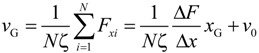
(1)
where, assuming conservative force and thermal fluctuations, we can apply:

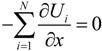
(2)

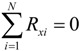
(3)


The term Δ*F*/(*N*Δ*x*) represents the ratio of the external force on the mass center to the displacement and ν_0_ is the initial velocity at the entrance. ζ is the friction coefficient fitted to represent the property of ssDNA and results in 4.68 × 10^−12^ kg/s. In Figure 6, the slopes of the plotted data in the initial portion of each graph are respectively 9.09 × 10^3^, 9.10 × 10^3^, and 9.80 × 10^3^ s^−1^ for the 30 × 30, 40 × 40, and 50 × 50 nm^2^ nanopores, giving an average value of 9.33 × 10^3^ s^−1^. In this region, the increment in which beads enter the nanochannel is almost constant despite the different channel cross-sections. In addition, when Δ*F*/Δ*x* is primarily due to the electric force in the nanochannel, we can write Δ*F* = *QE*_channel_Δ*N*, meaning that the change in the force is governed by the increase in the number of beads entering the channel under the almost uniform electric field. Equation (1) can then be replaced by:

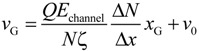
(4)
using *E*_channel_ approximated by the electric field at the center of the nanochannel as listed in Table 2. For the three cases, the values of Δ*N*/Δ*x* are 1.30 × 10^9^, 9.64 × 10^8^, and 7.45 × 10^8^ m^−1^for the 30 × 30, 40 × 40, and 50 × 50 nm^2^ nanopores, respectively (Table 2). A charged bead in an *E*_channel_ field generates *QE*_channel_ such that the displacement, Δ*x*, of the mass center related to each bead increment is proportional to *E*_channel_ and this explains why d*v*_G_/d*x*_G_ is almost constant for all three cross-sections. In the following region, when the mass center approaches *x*_G_ = 500 nm, there are obvious differences in velocity between the three cases. At this point, some beads are already in the nanopore. Subsequently, the velocity shows a moderate increase and appears to reach a terminal velocity when the center of mass passes through the nanopore. At this stage, the beads in the nanopore are driven forward due to the strong electric field and simultaneously experience counteracting force, being pushed back by the leading portion of the chain and pulled by the following portion. Since small nanopore cross-sections produce a strong driving force, the confinement in this region also gives rise to the counteraction including the entropic force and polymer–wall interactions. In this region, ν_G_ can be represented as:

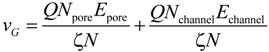
(5)
where *N*_pore_ and *N*_channel_ are the number of beads in the nanopore and nanochannel, respectively, and *E*_pore_ is the electric field strength in the pore. Here, the ssDNA chain is usually stretched and rarely collides with the wall as it passes through the interface between the nanochannel and the nanopore, and so the counteracting force is negligibly small compared to the other terms. As shown in Figure 5d and Table 2, the average number *N*_pore_ of beads in the nanopores is determined from the distributions. The remaining beads are in the nanochannel, such that *N*_channel_ = *N* − *N*_pore_. Using the electric field *E*_pore_ at the center of the nanopore and *E*_channel_, *v*_G_ in Equation (5) results in 3.54, 4.41, and 5.49 mm/s for the 30 × 30, 40 × 40, and 50 × 50 nm^2^ cross-section nanopores, respectively. These theoretically derived values are in good agreement with the simulations shown in Figure 6. Particularly, in the 30 × 30 nm^2^pore, the rapid change in curvature of the plot occurs at an *x*_G_ value of approximately 500 nm, indicating that the translocation process immediately reaches a steady state condition as the nanopore works to pump beads into the *trans* channel. In contrast, in the other pores, more moderate transitions of the velocity are observed and apparent transition points cannot be determined. As a result, the terminal velocities approach the theoretical values. Our data indicate that folded configurations of ssDNA chains in large cross-section pores cause moderate increases in the velocity of the mass center, and this results in high terminal velocities. In other words, our results explain why electrophoretic mobility decreases during transport through a confined space embedded in the fluidic channel [17,26,58]. From the viewpoint of molecular sequencing, increased knowledge of changes in the velocity and suppression of excessive increases in this velocity are desirable when attempting to ascertain details concerning the configuration changes of polymer molecules.

## 3. Langevin Dynamics Simulations of Polymer Chain Motion

A Langevin dynamics simulation was applied to investigate the behavior of a polymer chain passing through a three-dimensional nanopore embedded in a nanochannel, where the presence of solvent molecules could effectively be treated as a random force acting on the coarse-grained polymer molecule [16,45,46]. In the present model, strong effects of intramolecular interactions on the inertial force were coarse-grained and the kinetics of ssDNA were mainly affected by external electric fields. In such a case, the behavior of a particle can be expressed by an over-damped Langevin equation [16,45,46]:

ζ*_i_***v***_i_* = −*U_i_* + **F***_i_* + **R***_i_* (6)

where ζ*_i_* is the friction coefficient of the *i*th particle, −∇*U_i_* is the conservative force, including interactions between particles, and **F***_i_* denotes the external electrostatic force, such that **F***_i_* = −*Q_i_*ϕ, where *Q_i_* is the electric charge on the polymer molecule. For the purposes of a three-dimensional simulation, the electric potential, ϕ, in a rectangular nanofluidic channel was analyzed by solving for the Laplace equation ^2^ϕ = 0 with Neumann boundary conditions **n**·ϕ = 0 at the sidewall surfaces, where **n** was the surface normal vector, and with constant electric potentials at both ends of the channel. The FEM (Femtet^®^, Murata Software Co., Ltd., Tokyo, Japan) [53] was employed to solve for the electric potential. **F***_i_* was calculated by averaging the gradient of ϕ around each position [46]. In Equation (6), the random force **R***_i_* satisfied the fluctuation-dissipation theorem, such that:

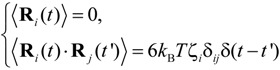
(7)
where *k*_B_ was the Boltzmann constant, *T* was temperature, δ*_ij_* was Kronecker’s delta and δ(*t* − *t*') was the Dirac delta function where *t* and *t*' were time. In this study, we focused on ssDNA and developed a bead-spring model for use in the Langevin dynamics simulations. Details of our coarse-grained model were also described in previous studies [16,45,46]. In order to model a ssDNA consisting of 48,000 nucleotides (48 knt), neighboring beads were connected with a harmonic spring [46]:

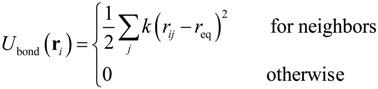
(8)
where *k* was the spring constant and *r_ij_* was the distance between the *i*th and *j*th particles. The equilibrium distance, *r*_eq_, between each connected pair of beads was defined as *r*_eq_ = α*r*_nt_*N*_nt_/*N*, where α was a variable parameter, *N*_nt_ was the number of nucleotides, and *N* was the number of beads. The equilibrium distance between the nucleotides in ssDNA is known to be *r*_nt_ = 0.43 nm [59] and so, applying an α value of 0.847 [16], *N*_nt_ = 48,000, and *N* = 400, we obtained *r*_eq_ = 43.7 nm. The above value for the parameter *α* was selected so as to properly replicate the radius of gyration (*R*_g_) [60] of ssDNA, as well as the diffusion coefficient and electrophoretic mobility. The harmonic spring constant was calculated as *k* = *k*_B_*T*/χ^2^, where *T* was set to 300 K and a χ value of 0.1σ was applied for thermal fluctuations based on previous studies [45], where σ was a Lennard–Jones parameter described below. Interactions between two beads, or between a bead and a channel wall, were represented by the Lennard–Jones potential, *U*_LJ_, taking into account the volume exclusion effect [46]:

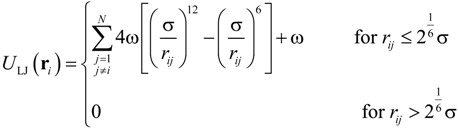
(9)
where σ was the characteristic length of ssDNA, and ω was the energy well-depth. A mirror reflection was assumed, meaning that the repulsive force from the wall effectively worked only along the direction perpendicular to the surface. The length parameter, σ, was determined from the persistence length of ssDNA necessary to reproduce the volume effect, such that σ = 5 nm [59]. *U*_LJ_ was applied to non-adjacent beads and ω was set to *k*_B_*T* [16,46]. For the purposes of volume exclusion, the potential was truncated at to allow for purely repulsive interactions between the beads. The term ζ*_i_* in Equations (6) and (7) was evaluated based on experimental measurements of the ssDNA diffusion coefficient, *D_i_*, according to *N*ζ*_i_D_i_* = *k*_B_*T* [52]. Applying *N* = 400, *D_i_* = 2.21 × 10^−12^ m^2^/s, and *T* = 300 K, ζ*_i_* was determined to be 4.68 × 10^−12^ kg/s for each bead. Considering the existence of counterions around the ssDNA, the effective charge of an individual bead could be calculated according to *Q_i_* = ζ*_i_*μ*_i_* = μ*_i_k*_B_*T*/*ND_i_* [52]. Thus, based on the experimental value of μ = 2.84 × 10^−8^ m^2^/Vs [52], a *Q_i_* value resulted in −0.83*e* per bead (consisting 120 nt), where *e* is the elementary charge. This value was determined in terms of electrophoretic mobility of the coarse-grained ssDNA including counterions and thus, it might underestimate the monomer charge previously known [58,61]. In order to verify this quantity, we performed Langevin dynamics simulations for the ssDNA model in free solution.

The overall structure of the fluidic channel, including the reservoirs outside the nanochannel, was taken into account in the preliminary analysis, as shown in Figure 3a. There was a reservoir of 2.5 × 4.0 × 0.5 μm (length × width × height) on either side of the nanochannel and the electrodes were 2.5 μm from the nanochannel entrance. The electric potentials at the electrodes were set to −0.400 and 0.400 V at the *cis* and *trans* sides, respectively, based on the experimental conditions summarized in Table 1. Additionally, the Laplace equation was solved in the nanochannel and nanopore with a fine resolution of 10 nm.

At equilibrium, *R*_g_ was maintained in the vicinity of 300 nm, such that *R*_g_^2^ was approximately equal to the product of the persistence length and the contour length [59,62]. Stable configurations such as this were employed as initial conditions for the simulations. The center of mass of the ssDNA was initially placed at a distance equivalent to *R*_g_ from the entrance of the nanochannel, as presented schematically in Figure 1a. In the next stage, the nonuniform electric field resulting from the FEM analysis was applied and the trajectories of the ssDNA were tracked. Equation (5) was integrated using the Euler algorithm with time steps of Δ*t* = 10 ps [46].

## 4. Conclusions

In this study, we investigated the electrostatic potentials in nanopores embedded in a rectangular nanochannel. We obtained considerable agreement in the electric field strengths on the order of 10^6^ V/m compared with previously published data [54]. Induction of strong electric fields in the narrowest space due to the connections of different-sized channels was confirmed [55]. Using such electric fields, we performed Langevin dynamics simulations by applying a coarse-grained model of ssDNA. The present model replicated the diffusion coefficient and electrophoretic mobility of long ssDNA, which allowed us to treat electrokinetic transport phenomena in the actual time and spatial scales. It was found that a nanoscale cross-sectional area was important with respect to uncoiling long-chained ssDNA molecules in a strong electric field and, as a result, reducing the translocation speed of the molecules. By adjusting the nanopore size, the quantity of ssDNA chains in the pore region can be constrained at a constant number, effectively producing a terminal velocity. With regard to the aim of obtaining single-molecule detection, this study suggests a preferred structure for nanofluidic channels.
